# Assessment of mouthguards worn by Irish children playing contact sports: an observational cross-sectional cohort study

**DOI:** 10.1007/s40368-022-00763-1

**Published:** 2022-11-17

**Authors:** Elaine Shore, Anne C. O’Connell

**Affiliations:** Division of Public and Child Dental Health, Dublin Dental University Hospital, Trinity College, Dublin, 2 Ireland

**Keywords:** Dental trauma, Paediatric, Protection, Mouthguard, Thickness, Retention, Quality

## Abstract

**Purpose:**

Traumatic dental injuries occur during participation in sports. Prevention of these injuries by wearing a mouthguard (MG) is desirable, especially in a paediatric population. There are several types of MG available, and their effectiveness depends on device design. The aim of this study was to examine the features of MG worn by children playing a contact sport (Gaelic football), and to determine whether these MG fulfilled recommendations for adequate dentoalveolar protection.

**Methods:**

A cross-sectional observational cohort study design was developed. Dentists were trained and calibrated in assessing MGs, both qualitatively (retention, extension, integrity) and quantitatively (thickness). A convenience sample of male and female subjects aged 9–16 years was selected for MG assessment via their sports clubs. Data were collected anonymously and analysed using descriptive and comparative statistics.

**Results:**

One hundred and six children presented with their MG for assessment. Two-thirds were wearing mouth-formed MG (*N* = 71, 66.96%). Only four were wearing custom MG (3.77%). Most MG had inadequate retention (*N* = 86, 81.13%) and labial extension (*N* = 89, 83.96%), with a significant relationship between MG type and retention. Mouthguard thickness varied widely at each site. Mouth-formed MG were significantly thicker than both stock and custom MG.

**Conclusions:**

Mouth-formed MG were the most common type followed by stock MG. Most MG did not have appropriate retention or labial extension. Parents and coaches need to be aware of MG features that maximise protection. If mouth-formed MG are provided in this age group, education on how to adapt them is essential. Paediatric dentists should check MG routinely for appropriate fit.

## Introduction

Traumatic dental injuries (TDI) result from accidental forceful impact to the teeth, mostly affecting maxillary central incisors (Andersson 2013). Long-term consequences of TDI (pulp necrosis, root resorption, and tooth loss) can be complex and costly to manage over a lifetime (Andersson 2013; Bani‐Hani et al. 2020), and these injuries are associated with poorer oral health-related quality of life (OHRQoL) (Zaror et al. 2018).

Sports-related injuries are responsible for 10–39% of all TDI in children (Newsome et al. 2001). Prevention of TDI would reduce the burden of care, including psychological and financial cost, to the individual, public dental services, and insurance companies. The use of personal protective equipment is increasingly being mandated or recommended by sporting organisations worldwide, especially in contact sports (Sigurdsson and Cohenca 2018). Mouthguards are most widely used to reduce the incidence of sports-related TDI, and are always recommended after a TDI to prevent re-injury.

A mouthguard (MG) is defined as “a resilient device or appliance placed inside the mouth to reduce oral injuries, particularly to teeth and surrounding structures” (Newsome et al. 2001). Mouthguards reduce the incidence and severity of sports-related TDI by increasing the surface area over which impact forces are applied to the dentoalveolar complex, thereby mitigating the forces applied to teeth (Sigurdsson and Cohenca 2018).

There are three types of MG: stock, mouth-formed, and custom MG (Table 1).

**Table 1:** Characteristics of the various mouthguard types

Mouthguard type	Advantages	Disadvantages
Stock	Most inexpensive type	Predetermined sizes; cannot be modified to fit (Sigurdsson and Cohenca 2018)
		Fit loosely, must be held in place by clenching (Gawlak et al. 2015)
		Impede speech and breathing (Gawlak et al. 2015)
Mouth-formed (“boil-and-bite”)	Widely available, relatively inexpensive, and tend to fit better than stock MG (Gawlak et al. 2015; Sigurdsson and Cohenca 2018)	Relatively thin over labial and occlusal surfaces (Patrick et al. 2005)
		May inadequately cover posterior teeth (Kuebker et al. 1986)
Custom	Improved fit and thickness	Most expensive type
	More comfortable to wear (Gawlak et al. 2015)	Require attendance with a dental professional for 1–2 appointments

Stock MG (SMG) are ready-to-wear, and are considered the least protective MG type as their lack of adaptation to the dentoalveolar tissues may increase the likelihood of the MG being dislodged in the event of an injury (Patrick et al. 2005). Mouth-formed MG (MFMG) are fabricated from a thermoplastic material and are modified to fit the wearer by heating the device in boiling water, and then moulding it using intraoral biting and sucking pressures (Sigurdsson and Cohenca 2018). Dentists are rarely involved in evaluating the fit of these mouthguards for their patients. Custom MG are made from individual impressions or digital scans of dental arches using models and either vacuum- or pressure-forming techniques. (Gawlak et al. 2015). These MG have improved fit and more uniform thickness, so they are more comfortable to wear (Gawlak et al. 2015).

An effective MG must limit the force transmitted to the maxillary teeth and supporting tissues through optimal thickness, shock absorption, and retention (Guerard et al. 2017). Evidence from the literature for the various features of an ideal MG is summarised in Table 2.

**Table 2:** Criteria for ideal mouthguard fabrication

Coverage	Cover all maxillary teeth to the distal aspect of the second permanent molars (Scott et al. 1994), or to the most posterior erupted tooth in children
Thickness	Labial surface of central incisors: 3–4 mm (Maeda et al. 2008; Verissimo et al. 2016) Occlusal surface of posterior teeth: 2–3 mm (Maeda et al. 2008; Murakami et al. 2008) Incisal edge of anterior teeth: 4 mm (Westerman et al. 2002) Palatal: 1 mm (Scott et al. 1994)
Labial extension	2 mm short of vestibular reflection, smooth, and rounded in cross section (McClelland et al. 1999)
Palatal extension	Just beyond the cervical margin of the palatal surface of the teeth, smooth, and tapered in cross section (Karaganeva et al. 2019; Maeda et al. 2006)
Occlusion	Balanced occlusion (McClelland et al. 1999; Takeda et al. 2008; Veríssimo et al. 2017)

The performance of and protection afforded by the MG depends on the design and materials used in fabrication. Mouthguards must fit properly and be well retained (Scott et al. 1994) while allowing players to breathe freely during wear (Collares et al. 2014; Maeda et al. 2006). The material used to fabricate MG should be non-irritant and easily cleaned (Scott et al. 1994) and should be thick enough to reduce stress and strain on the dentition in the event of an impact to the teeth, thereby reducing the risk of injury (Verissimo et al. 2016). Mouthguards are most commonly fabricated from ethylene vinyl acetate (EVA).

Gaelic football is an amateur contact sport in Ireland popular among people of all ages across the country. Since 2014, MG have been mandatory for male and female Gaelic football players of all ages during training and competition (Gaelic Athletic Association 2016; Ladies Gaelic Football Association 2013). The requirements for these MG state only that the player must feel that it fits properly, and it must carry the CE mark (a European Union requirement for health and safety of all products sold in the EU) (European Union 2021; Gaelic Athletic Association 2016); . There is no stipulation about the type or design of MG. Previously, we reported that compliance with the mandated MG rules varied among 9–16-year-old GAA players, with reduced adherence to the rules in older children (Shore and O’Connell 2021). The aim of this study was to examine the features of MG being worn by a sample of children playing Gaelic football in Ireland, and to determine whether these MG fulfilled recommendations for adequate dentoalveolar protection (Table 2).

## Materials and methods

Ethical approval was obtained from the Trinity College Dublin Faculty of Health Sciences Research Ethics Committee (2nd November 2018, reference 180901). Permission was granted by the Gaelic Athletic Association (GAA) and Ladies’ Gaelic Football Association (LGFA) to conduct this research among Gaelic football players. A cross-sectional observational cohort design was employed.

A research team of dentists were recruited, trained, and calibrated in assessing the type, fit, retention, and thickness of MG. There were no previous studies upon which to base a sample size calculation, so the aim was to examine 30–50 children per club. A convenience sample of 14 Gaelic football clubs was invited to participate in the study. Male and female Gaelic football players aged 9–16 years who were willing to participate and whose parents provided informed consent were included in this study.

The research team visited clubs during Gaelic football training sessions on dates agreed upon with each club. Players and their parents presented themselves to the research team. The research team worked in pairs. One dentist assessed the MG, while the other recorded their findings. The examination was performed in a clean environment with a portable chair, artificial light, and callipers (IGaging® 8″ Digital Outside Callipers, California, USA). Strict infection control measures were in place for the examination.

**Fig. 1:**
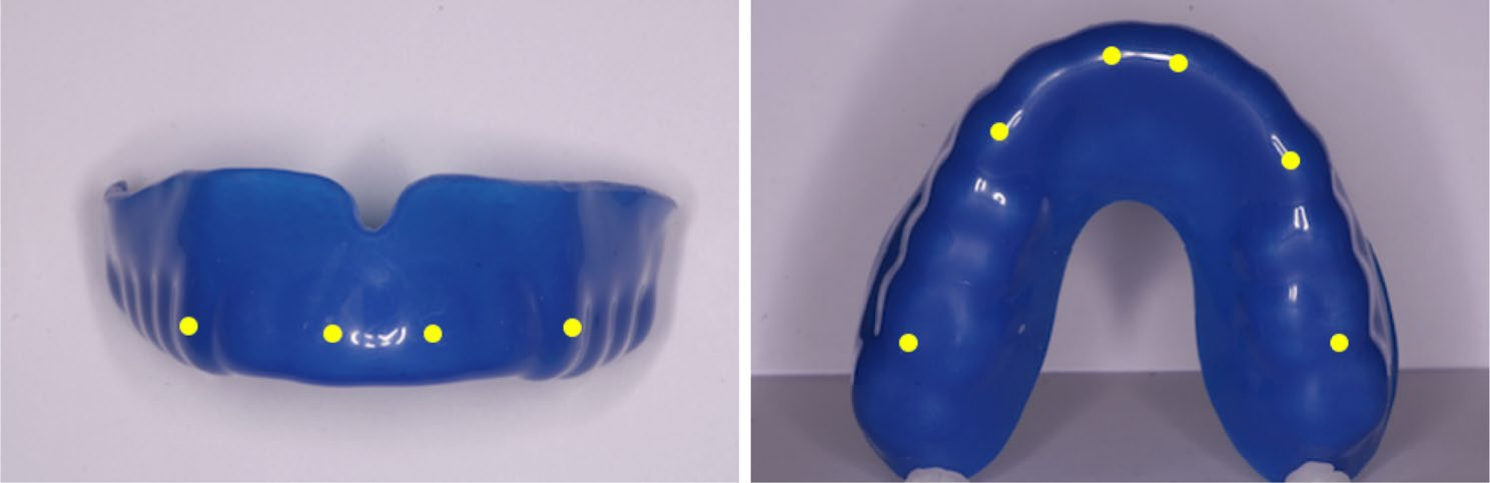
Measurement locations on mouthguards

Mouthguard thickness was measured at labial and occlusal surfaces of 6 teeth (upper central incisors, canines, and first permanent molars, Fig. 1) using a modified external gauge callipers correct to 0.1 mm (IGaging® 8″ Digital Outside Callipers, California, USA).

The type of MG, labial and palatal extension, and smoothness and deformation were easily determined by visual examination, and were scored as adequate/inadequate. Retention was assessed by ease of dislodgement by light finger pressure anteriorly, and by asking each subject to open widely while wearing the MG. Occlusion was determined by observation of bilateral simultaneous contacts, while subjects clenched their teeth together on the MG.

Data were collected anonymously, tabulated using Excel (Microsoft Corporation, 2018), and exported to SPSS (Statistical Package for Social Sciences, version 26, IBM Corp., Chicago IL, USA) for analysis. Statistical tests included Kolmogorov–Smirnov test for normality, descriptive statistics (mean and standard deviation; median and interquartile range), and comparative statistics [chi-squared (χ^2^), Kruskal–Wallis test (H)]. Inter- and intra-rater agreement was measured using the intra-class correlation coefficient (ICC) and Cohen’s kappa (κ). Results were reported with a 95% confidence interval and a significance level of 5%.

## Results

Four GAA clubs accepted the invitation to take part in the study. Data collection was completed between September 2019 and January 2020. Plans for further data collection were suspended thereafter due to the COVID-19 pandemic. Parental consent was obtained for 121 children aged 9–16 years playing Gaelic football from the four clubs. Only 106 children (87.6%) brought their MG with them for examination.

Inter-rater agreement for qualitative variables (type, retention, and extension of MG) was high (κ > 0.8). Inter-rater agreement for MG thickness was also high (ICC for labial thickness = 0.953; ICC for occlusal thickness = 0.861). Intra-rater reliability for labial thickness measurements was high for all examiners (> 80%). Intra-rater reliability for occlusal thickness measurements was high for Examiners 1 and 2 (≥ 80%), but was 47.5% for Examiner 3.

**Fig. 2:**
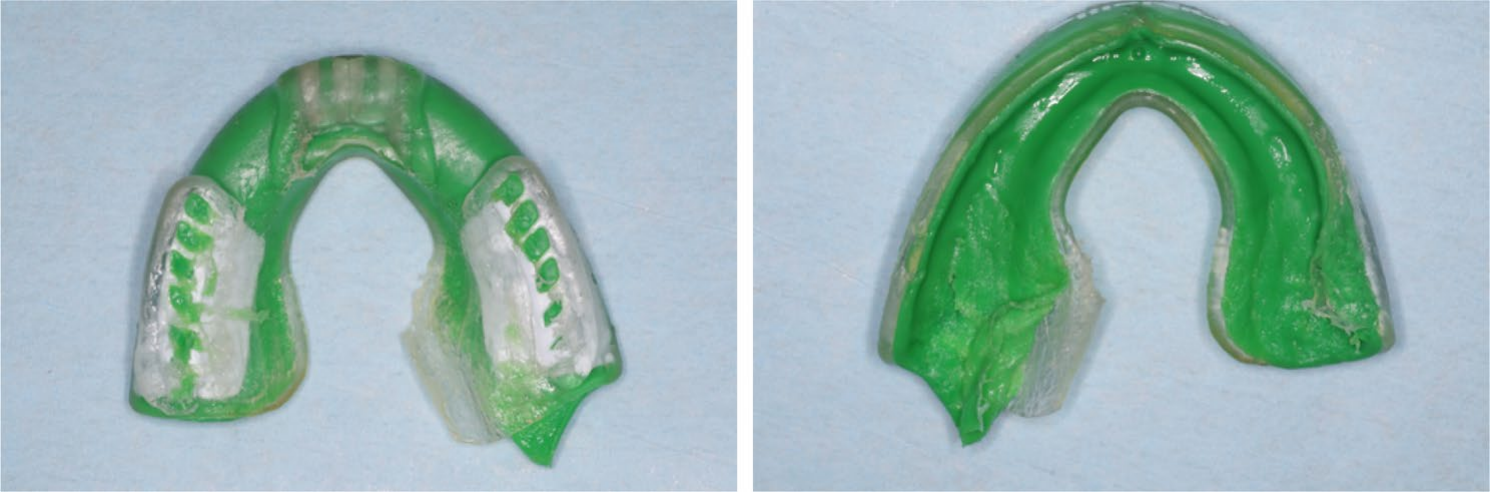
Mouth-formed MG with considerable deformation on the posterior palatal flange

Two-thirds of the sample wore MFMG (*N* = 71, 66.98%); only four participants (3.77%) wore CMG. The fit and thickness of all MG were assessed (Table 3). Most MG (*N* = 86, 81.13%) had inadequate retention (were easily dislodged during examination) and/or insufficient labial extension according to criteria outlined in Table 2 (*N* = 89, 83.9%). Approximately half of the MG had sufficient occlusal coverage (*N* = 57, 53.77%), appropriate balanced occlusion (*N* = 57, 45.19%), and an intact surface (*N* = 54, 50.94%). Forty-seven MG (44.34%) displayed shape deformation such as in the example seen in Fig. 2.

**Table 3:** Frequencies of fit-related characteristics of mouthguards

	Adequate (*N*, %)	Inadequate (*N*, %)	Total
Retention	20 (18.87%)	86 (81.13%)	106
Labial extension	17 (16.04%)	89 (83.96%)	106
Palatal extension	64 (60.37%)	42 (39.62%)	106
Occlusal coverage	57 (53.77%)	49 (46.23%)	106
Balanced occlusion^a^	47 (45.19%)	57 (54.81%)	104
Border smoothness^b^	45 (46.88%)	51 (53.13%)	96
Mouthguard surface	54 (50.94%)	52 (49.06%)	106

^a^Balanced occlusion: missing data for two subjects, excluded from analysis

^b^Borders: missing data for ten subjects. Excluded from analysis

Most SMG (*N* = 27, 87.1%) and MFMG (*N* = 59, 83.1%) had inadequate retention. The relationship between MG type and retention was significant (χ^2^ = 18.10, *p* < 0.001; Table 4).

**Table 4:** Relationship between mouthguard type and fit characteristics

Variable	Mouthguard type					
	Stock MG *N* (%)	Mouth-formed *N* (%)	Custom	Total *N* (%)	χ^2^	*p*
*Retention*						
Inadequate	27 (87.1)	59 (83.1)	0 (0.0)	86 (81.1)	18.100	< .001*
Adequate	4 (12.9)	12 (16.9)	4 (100.0)	20 (18.9)		
*Labial extension*						
Inadequate	28 (90.3)	59 (55.7)	2 (50.0)	89 (84.0)	4.397	.111
Adequate	3 (9.7)	12 (16.9)	2 (50.0)	17 (16.0)		
*Palatal extension*						
Inadequate	15 (48.4)	27 (38.0)	0 (0.0)	42 (39.6)	3.696	.158
Adequate	16 (51.6)	44 (62.0)	4 (100.0)	64 (60.4)		
*Occlusal coverage*						
Inadequate	14 (45.2)	34 (47.9)	1 (25.0)	49 (46.2)	0.818	.664
Adequate	17 (54.8)	37 (52.1)	3 (75.0)	57 (53.8)		
*Balanced occlusion*^*a*^						
Inadequate	19 (63.3)	37 (64.9)	1 (25.0)	57 (54.8)	2.423	.298
Adequate	11 (36.7)	33 (47.1)	3 (75.0)	47 (45.2)		
*Border smoothness*^*b*^						
Inadequate	17 (58.6)	34 (53.1)	0 (0.0)	51 (53.1)	3.752	.153
Adequate	12 (41.4)	30 (46.9)	3 (100)	45 (46.9)		

Abbreviations: χ^2^ = chi-squared test, *CI*  confidence interval

*p < 0.05, 95% CI

^a^Balanced occlusion: missing data for two subjects. Excluded from analysis

^b^Borders: missing data for ten subjects. Excluded from analysis

Mouthguard thickness was measured for all central incisor, canine, and first permanent molar sites as indicated in Fig. 1. The value for MG thickness was not normally distributed (*p* < 0.001) and there was a wide range of measurements for each site. The median labial MG thickness at the central incisor site was 4.05 mm (interquartile range = 3.51–4.55 mm). The median posterior occlusal thickness, measured at the first permanent molar site, was 3.9 mm (interquartile range = 3.1–6.4 mm), and the median incisal edge thickness at the central incisors was 4.53 mm (interquartile range = 2.91–7.3 mm).

Mouth-formed MG were significantly thicker than both SMG and CMG (H = 20.409, *p* < 0.001; Table 5) at the central incisor site. Custom MG were significantly thinner than both SMG and MFMG on the occlusal surface of the first permanent molars (Table 5).

**Table 5:** Association between mouthguard type and thickness

Mean thickness (mm)	Mouthguard type mean, sd (mm)			Kruskal–Wallis H	*p*
	Stock	Mouth-formed	Custom		
Central incisor, labial	3.67 ± 0.65	4.58 ± 1.26	3.18 ± 0.43	20.41	< .001*
Central incisor, incisal	4.08 ± 2.64	5.18 ± 2.27	2.86 ± 0.62	3.91	.142
First molar, occlusal	4.64 ± 2.01	4.77 ± 1.94	2.58 ± 0.41	6.55	.038*

Abbreviations: *sd* standard deviation, *CI*  confidence interval; *p < 0.05, 95% CI

## Discussion

Mouthguards are recognised by sporting organisations as an essential piece of personal protective equipment. Prevention of sports-related TDI is crucial in a child and adolescent population as there are lifelong implications of suffering an injury to permanent teeth at a young age. Existing data on types of MG being worn come from questionnaire-based studies of sports participants and/or parents of children playing sports (Kroon et al. 2016; O’Malley et al. 2012; Shore and O’Connell 2021). This observational study is the first to explore the reality of MG use by children playing contact sport, by investigating the type, quality and fit of MG being used in a real-time field setting. It is important to compare the quality of MG being worn to the criteria for an ideal mouthguard (Table 2). These findings can be used to educate those involved in sports and dental colleagues to maximise protection of teeth during sporting activities using appropriate mouthguard design.

The study was conducted within Gaelic football clubs as the governing bodies introduced rules in 2014 requiring the wearing of MG for all players of all ages during Gaelic football training and competitive events (Gaelic Athletic Association 2016; Ladies Gaelic Football Association 2013). As this is a highly popular contact sport in Ireland, it was anticipated that a meaningful number of players and MG could be accessed for quality assessment.

Unfortunately, the final sample size was smaller than expected as challenges were encountered in recruiting participants, and COVID restrictions caused cancellation of all sporting activities, and therefore, data collection was suspended early.

Consent was obtained for the participation of 121 children in the study, but 15 of these had left their MG at home, so the final sample was of 106 children with their MG. Dentists assessed the MG in real time during a routine training session. Most children (*N* = 71, 66.98%) wore a mouth-formed MG. Only four (3.8%) wore a custom MG; this was much lower than anticipated.

Mouthguard fit was judged based on MG retention, extension, and occlusion (Table2). Retention is determined by the accuracy of fit of the MG material around the dentoalveolar structures, and assessed by ease of dislodgement. Over 80% of the MG in this study had poor retention, compromising their ability to protect the teeth in the event of a TDI as they may be dislodged instead of absorbing and dissipating impact forces. Poor retention may also limit the ability to communicate, which is essential in a team sport. The authors previously reported that 39.2% (*N* = 47) of the children in the same sample reported difficulty speaking while wearing their MG (Shore and O’Connell, 2021).

There was a significant relationship between MG type and retention. Most SMG (83%) had inadequate retention, likely because these MG cannot be adjusted in any way (Sigurdsson and Cohenca 2018). Eighty-seven per cent (*N* = 59) of MFMG had inadequate retention, suggesting that parents and children in this sample were not properly performing the moulding procedure for these MG. The four custom MG in this study all had adequate retention; definitive conclusions cannot be drawn due to the small sample size.

Most MG (*N* = 89, 83.86%) had inadequate labial extension. This is concerning as MG should cover the teeth and supporting dentoalveolar structures to provide maximum protection. Sixty-four MG (60.37%) had adequate palatal extension. Once the MG material engages the palatal cervical undercut properly, the palatal flange is minimally important for MG retention, but it does affect comfort and wearability (Karaganeva et al. 2019; Maeda et al. 2009, 2006).

Mouthguards should cover all erupted maxillary teeth (Scott et al. 1994). Only 53.77% (*N* = 57) of the MG in this study had adequate occlusal coverage. This is not surprising given the fact that most of the MG were either SMG or MFMG, which are fabricated in predetermined sizes. A previous study reported that 85% of adult male basketball players’ MFMG had inadequate occlusal coverage (Kuebker et al. 1986). The current study indicated better occlusal coverage; it is possible that such MG fit children and adolescents better than adults.

Balanced occlusion, especially anteriorly, is important for impact force absorption and dissipation (Takeda et al. 2008; Veríssimo et al. 2017). Less than half of the MG in this study (*N* = 47, 45.19%) fulfilled this criterion. No clinical studies were found in children to associate the importance of balanced occlusion and injury prevention. This should be explored further in future clinical research, as previous investigations have been laboratory-based.

Mouthguard surfaces should have no perforations or other disruptions to optimise comfort, wearability, and hygiene (Almeida et al. 2018). Approximately half of the MG had sharp or rough borders (*N* = 51, 53.13%) and/or unsatisfactory surface integrity (*N* = 52, 49.06%). Wearing MG while playing sports may be associated with the development of oral soft-tissue lesions (Glass et al. 2009). Only one child in this cohort reported not wearing MG due to discomfort (Shore and O’Connell 2021). The current study did not include a soft-tissue examination; this would be a useful area for future investigation.

Almost half of the MG in this study (*N* = 47, 44.34%) were deformed in some way; some subjects had bitten on their MG so much as to flatten the buccal flanges completely (Fig. 2). Deformation of the MG can occur due to time, storage conditions, or when players engage in habitual inappropriate chewing of their MG (Del Rossi et al. 2007). These conditions alter MG thickness and fit over time, resulting in a less-effective and less-protective device (Del Rossi et al. 2007).

Thickness of MG chosen and worn by children playing sports has not previously been assessed in an observational field study. Mouthguard thickness has been investigated via in vitro or finite-element analyses. The ideal MG thickness for the various measurement sites has been established by the materials’ science literature based on the adult dentition (Table 2). There was a wide range of thickness values for each measurement site in this study, reflecting the different types of MG observed. It was difficult to identify reproducible measurement sites on each MG due to the different MG design, border deformation, and individual variation in anatomical landmarks. There was a low intra-rater agreement for one examiner in occlusal thickness measurements only. A decision was made not to reduce examiners throughout the study due to the number of subjects to be assessed at any given time, as well as the variety of dates and times agreed with the clubs. As a clinical observational study in a real-life setting, the data and results presented in this paper are valuable despite these variations in some measurements as it is the only study of its kind carried out to date.

The median labial and incisal edge thicknesses of each MG type were appropriate in this sample (Table 5). The median posterior occlusal thickness of each MG was greater than that suggested in the literature recommended to avoid the development of temporomandibular joint derangements (Maeda et al. 2008; Murakami et al. 2008).

Overall, MFMG were thicker than CMG, but the number of CMG was too low for statistical analysis (Table 5). These findings are in contrast to much of the existing literature comparing these MG types (Guerard et al. 2017; Park et al. 1994). The greater thickness of MFMG in this study may reflect individual variation in the moulding and self-adaptation process.

This observational study had a number of limitations. The overall sample size and the number of custom mouthguards presented for examination were lower than anticipated. It was difficult to identify reproducible measurement sites on each MG due to the variety of different MG types used and individual variation in anatomical landmarks. It would have been ideal to have reached excellent calibration for all measurement, but we accepted the lower intra-rater agreement for one of the examiners for occlusal thickness measurements.

There are no randomised clinical trials investigating the effectiveness of different MG types in protection from injury. Impact tests in vitro have shown that custom MG demonstrate greater shock absorption ability (Bemelmanns and Pfeiffer 2001) and result in fewer fractured teeth (Greasley et al. 1998) than MFMG. Custom MG also have improved occlusal stability and fit than MFMG (Gawlak et al. 2015; Hoffmann et al. 1999; Patrick et al. 2005). Considering the available evidence, a well-fabricated CMG of sufficient extension, retention, and thickness with balanced occlusion will offer improved protection than an MFMG. It would seem prudent for dentists and coaches to encourage the increased use of CMG for children playing contact sports and to avoid SMG. There is a role for dentists to advise on the suitability of the current mouthguard and to offer individual advice on the most appropriate MG for that child’s activities. This study has shown that most MFMG were poorly formed, identifying a need for education in the appropriate forming methods to parents. Dentists should include a mouthguard evaluation into a routine dental visit for children playing sports to maximise protection from sports-related dental injury.

## Conclusions

Given the limitations of the study, the following conclusions can be made:

- In this study, a variety of MG types were worn, with MFMG being the most popular type.
- The quality of MG worn by the participants was poor.
- Most MG had inadequate retention and labial extension, with variability in average MG thickness at each site. Average labial and incisal thicknesses were appropriate, while average occlusal thickness was greater than that recommended in the literature.
- MFMG were thicker than both SMG and CMG at all sites. If MFMG are necessary, players/parents should be shown how to properly mould these devices.
- There is a need for education regarding the criteria for a properly fitted MG.
- Paediatric dentists should promote the need for wearing appropriate MG and should evaluate quality and fit of mouthguards as part of the regular dental examination.

## Data Availability

The datasets generated during and/or analysed during the current study are available from the corresponding author on reasonable request.
